# Bacterial Quorum-Sensing Signal DSF Inhibits LPS-Induced Inflammations by Suppressing Toll-like Receptor Signaling and Preventing Lysosome-Mediated Apoptosis in Zebrafish

**DOI:** 10.3390/ijms23137110

**Published:** 2022-06-26

**Authors:** Hongjie Zhu, Zhihao Wang, Wenxin Wang, Yongbo Lu, Ya-Wen He, Jing Tian

**Affiliations:** 1 Zebrafish Model Research Center for Human Diseases and Drug Screening in Western China, School of Medicine, The College of Life Sciences, Northwest University, Xi’an 710069, China; zhu-hongjie@outlook.com (H.Z.); zhihaowang9264@gmail.com (Z.W.); wang-wenxing@outlook.com (W.W.); yongerbo@outlook.com (Y.L.); 2 State Key Laboratory of Microbial Metabolism, Joint International Research Laboratory of Metabolic & Development Sciences, School of Life Sciences & Biotechnology, Shanghai Jiao Tong University, Shanghai 200240, China

**Keywords:** diffusible signal factor (DSF), quorum-sensing signal, anti-inflammation, zebrafish model, Toll-like receptor signaling, lysosome-mediated apoptosis

## Abstract

Bacteria and their eukaryotic hosts have co-evolved for millions of years, and the former can intercept eukaryotic signaling systems for the successful colonization of the host. The diffusible signal factor (DSF) family represents a type of quorum-sensing signals found in diverse Gram-negative bacterial pathogens. Recent evidence shows that the DSF is involved in interkingdom communications between the bacterial pathogen and the host plant. In this study, we explored the anti-inflammatory effect of the DSF and its underlying molecular mechanism in a zebrafish model. We found that the DSF treatment exhibited a strong protective effect on the inflammatory response of zebrafish induced by lipopolysaccharide (LPS). In the LPS-induced inflammation zebrafish model, the DSF could significantly ameliorate the intestinal pathological injury, reduce abnormal migration and the aggregation of inflammatory cells, inhibit the excessive production of inflammatory mediator reactive oxygen species (ROS) content, and prevent apoptosis. Through an RNA-Seq analysis, a total of 938 differentially expressed genes (DEGs) was screened between LPS and LPS + DSF treatment zebrafish embryos. A further bioinformatics analysis and validation revealed that the DSF might inhibit the LPS-induced zebrafish inflammatory response by preventing the activation of signaling in the Toll-like receptor pathway, attenuating the expression of pro-inflammatory cytokines and chemokines, and regulating the activation of the caspase cascade through restoring the expression of lysosomal cathepsins and apoptosis signaling. This study, for the first time, demonstrates the anti-inflammatory role and a potential pharmaceutical application of the bacterial signal DSF. These findings also suggest that the interkingdom communication between DSF-producing bacteria and zebrafish might occur in nature.

## 1. Introduction

Inflammation is an essential physiological reaction process of the human immune system that can protect the body from stimulation and restore damaged tissue structure and function [1]. However, excessive and uncontrolled inflammatory responses can induce various chronic diseases and disorders, such as cancer [2], type 2 diabetes [3], cardiovascular disease, arthritis [4], autoimmune diseases, inflammatory bowel disease [5], and neurodegenerative disease. Thus, it is very important to correctly regulate the inflammatory response. The typical inflammatory response consists of inducers, sensors, mediators, and target tissues [1]. As a sensor, Toll-like receptors (TLRs) expressed by different immune cells, such as macrophages, dendritic cells, and mast cells, can induce the production of a variety of inflammatory mediators, such as cytokines, including interleukin (IL)-1β, IL-6, and tumor necrosis factor α (TNF-α) [6]. In addition, nuclear factor kappa B (NF-κB) and mitogen-activated protein kinase (MAPK) are also important inflammatory signals in the TLR pathway [7]. Lipopolysaccharide (LPS) is the major component of the outer wall of Gram-negative bacteria. It triggers the increase in pro-inflammatory cytokines, platelet-activating factors, prostaglandins, enzymes, and free radicals (such as nitric oxide), resulting in the inflammatory response of the myocardium, lungs, and intestines [8,9,10]. Thus far, LPS has been widely used in the research of inflammatory diseases and the construction of inflammatory disease models [11]. Therefore, screening agents that can regulate the production of these inflammatory cytokines, mediators, and inflammatory signals have attracted more and more attention in the development of anti-inflammatory drugs.

The diffusible signal factor (DSF) family is involved in an intriguing type of quorum-sensing signal molecules produced by diverse Gram-negative bacterial pathogens [12,13]. *cis*-11-methyl-2-dodecenoic acid was the first DSF signal found in the plant pathogen *Xanthomonas campestris* pv. *campestris* (*Xcc*) [14]. All DSF family signals are *cis*-2-unsaturated fatty acids containing fatty acid carbon chains of various lengths, a *cis* double-bond configuration, and side-chain composition [13,15]. DSF family signals have been detected in the bacterial species *Xanthomonas*, *Xylella fastidiosa*, *Stenotrophomonas maltophilia*, *Lysobacter enzymogenes,* L. *brunescens*, *Leptospirillum ferri philum*, L. *ferrooxidans*, *Burkholderia cenocepacia*, *Cronobacter turicensis*, and *Pseudomonas aeruginosa* [13,16]. These bacteria use the DSF family signal to sense the cell population and to function as a multi-cellular organism that executes a broad range of functions critical for bacterial survival and pathogenesis [12,13,16]. In the last decade, an enormous repertoire of DSF-mediated interspecies communication has been observed between DSF-producing bacteria and *Bacillus*, *Francisella novicida*, *Salmonella*, *Bdellovibrio bacteriovorus*, and *Candida albicans* [17]. For example, the addition of the DSF can inhibit the synthesis of extracellular polysaccharides (EPSs) and induce the dispersion of a biofilm, which has a potential anti-bacterial effect [17,18]. As a natural extract, the DSF is rarely applied to animals. Studies have shown that *cis* 2-hexadecenoic acid (c2-HDA), a member of the DSF family produced by *Xylella fastidiosa*, can repress the expression of the virulence gene of intestinal pathogenic *Salmonella* in a murine colitis model, thus, affecting its colonization of the mouse intestine [19]. However, the effects of the DSF on animal growth and development have not been studied.

The zebrafish (*Danio rerio*) has become a well-known animal model for the study of human diseases. Zebrafish have unique advantages, such as convenient feeding, a short development cycle, a large amount of eggs, and transparent and easy observation of embryos [20]. As a vertebrate, the zebrafish is similar to humans in tissue and developmental biology. Moreover, the zebrafish has an 87% similarity in genomes with humans, and more than 80% of disease proteins are conserved, which causes it to have a wide range of applications in disease modeling, phenotypic research, drug screening, and toxicology [21]. In addition, the temporal separation between innate and adaptive immune responses and the transparency of the embryonic stage, which allows for real-time visualization, are unique advantages in the study of innate immunity and inflammation [22]. The identification of zebrafish macrophage subtypes shows that the evolution of inflammatory cells is conserved from fish to mammals. Although zebrafish cytokines have a low amino acid sequence homology compared to human cytokines, their identification and characterization show that they have similar functions and structures [23]. The inflammatory responses of zebrafish can be induced with physical methods, such as cutting off the caudal fins and UV irradiation [24,25]. It has been reported that zebrafish embryos exposed to trinitrobenzene sulfonic acid (TNBS) and dextran sodium sulfate (DSS) can be used to establish a larval model of enterocolitis [26]. LPS has also been used to construct the zebrafish inflammation model. Soaking zebrafish larvae in an embryo culture medium containing LPS or microinjecting LPS into the egg yolk induce a series of inflammatory reactions [24,27], which can also be used to induce the inflammatory response of adult zebrafish [28]. Under LPS stimulation, zebrafish exhibit an increased level of reactive oxygen species (ROS), the infiltration of neutrophils, and the up-regulated expression of inflammation-related factors such as *il1b*, *il6*, and *tnfa* [21]. The zebrafish inflammation model induced with LPS has been widely used in the study of anti-inflammatory effects of small molecular compounds, various extracts, and traditional Chinese medicine (TCM) [21,24,29].

In this study, for the first time, we evaluated the effect of the DSF on the LPS-induced inflammatory response in a zebrafish model. The potential molecular mechanism of the DSF anti-inflammatory effect was investigated using a transcriptome profiling analysis and was further verified in zebrafish.

## 2. Results

### 2.1. The Developmental Effect of DSF on Zebrafish Embryos

**Figure 1:**
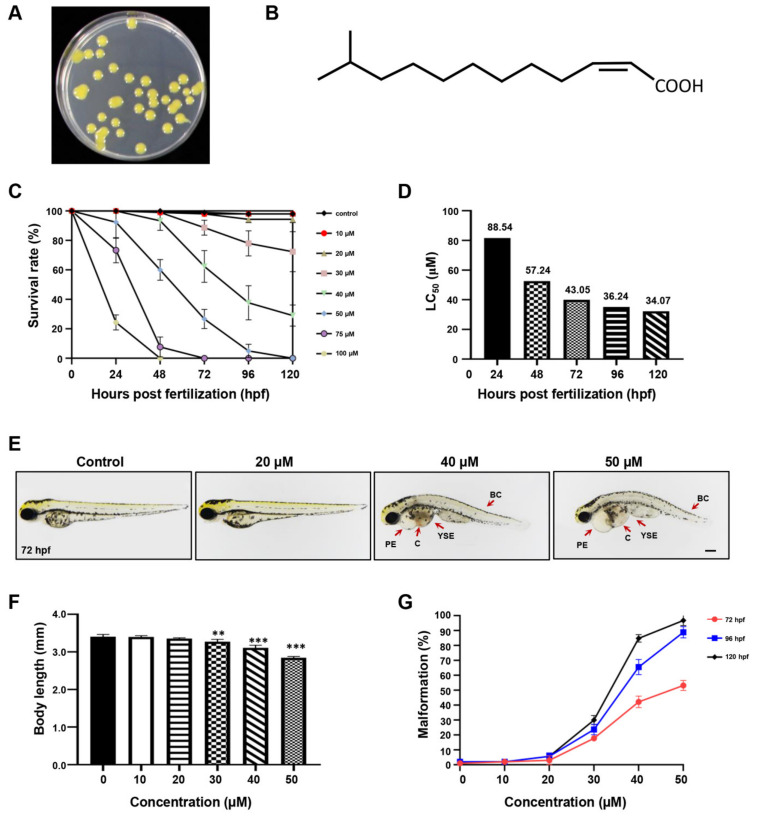
The effect of DSF on zebrafish embryos. (**A**) DSF producer *Xcc*. (**B**) The chemical structure of DSF. (**C**) The survival rate of embryos treated with different doses of DSF at 24, 48, 72, 96, and 120 hpf. (**D**) LC50 of DSF-treated embryos. (**E**) DSF-treated embryos (0, 20, 40, and 50 μM) revealed different malformed phenotypes at 72 hpf. PE, pericardial edema; YSE, yolk sac edema; C, congestion; BC, body curvature. (**F**) The body length was calculated at 72 hpf after embryos were treated with different doses of DSF. (**G**) Quantification of the malformation rate at 72, 96, and 120 hpf embryos after treatment with different doses of DSF. The LC_50_ was calculated with IBM SPSS 22 statistics. The values are represented as mean ± S.D. ** *p* < 0.01, *** *p* < 0.001. Scale bar, 200 μm.

The DSF cis-11-methyl-2-dodecenoic acid was purified from *Xanthomonas campestris* pv. *campestris* (Xcc) (Figure 1A). The chemical structure of the DSF is shown in Figure 1B. To investigate the potential developmental effects of the DSF on zebrafish, seven different concentrations of DSF (10, 20, 30, 40, 50, 75, and 100 μM) were applied to embryos, and the mortality was recorded at 24, 48, 72, 96, and 120 hpf. The DSF dose-dependently decreased the survival rate of embryos (Figure 1C). The LC_50_ of the DSF is shown in Figure 1D. At 72 hpf, with the concentration of the DSF increased (above 20 μM), embryos developed significant body deformities, including pericardial edema (PE), yolk sac edema (YSE), congestion (C), and body curvature (BC) (Figure 1E). The body length was decreased (Figure 1F) and the malformation rate was increased in a dose-dependent manner (Figure 1G). Taken together, the DSF resulted in zebrafish embryo developmental toxicity when embryos were exposed to a concentration of the DSF above 20 μM. Here, we chose 20 μM of the DSF for subsequent experiments to study the anti-inflammatory effect of the DSF in zebrafish.

### 2.2. DSF Restores LPS-Induced Intestinal Mucosa Injury in Zebrafish

**Figure 2:**
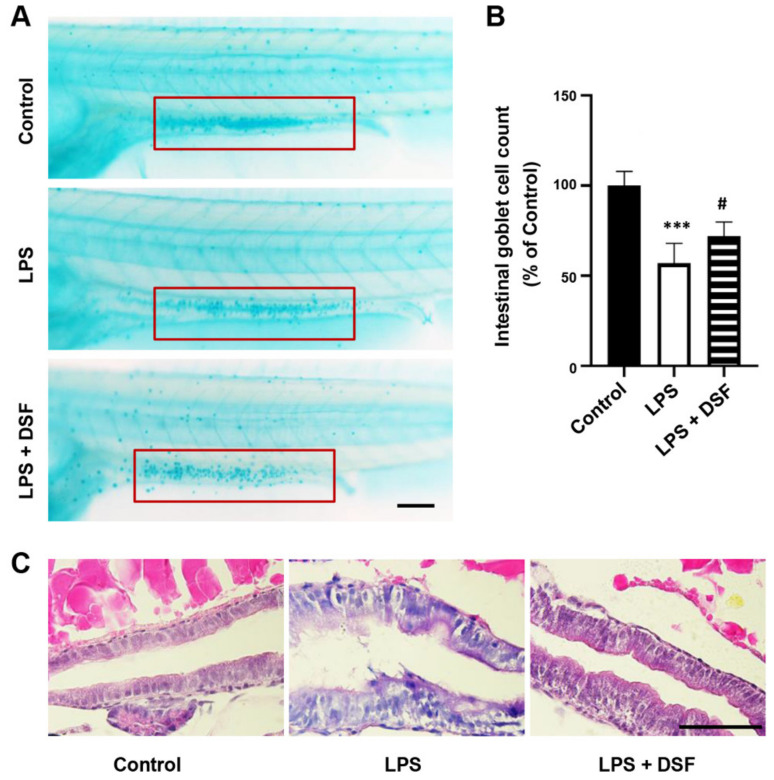
DSF ameliorated intestinal damage of zebrafish embryos after LPS-induced inflammation. (**A**) The goblet cells in zebrafish intestinal mucosa were stained with alcian blue (red boxes). (**B**) The intestinal goblet cells (red boxes) in A were enumerated. (**C**) The histopathological changes in the zebrafish intestine were detected with H&E staining. Data are represented as mean ± S.D. *** *p* < 0.001 vs. control group; ^#^ *p* < 0.05 vs. LPS group. Scale bar, 100 μm.

To investigate the anti-inflammatory effect of the DSF, LPS was used to establish a zebrafish inflammation model. The LPS-induced intestinal damage could be detected with alcian blue staining of goblet cells in the intestinal mucosa [30]. As shown in Figure 2A, compared with the control group, the number of goblet cells in the LPS-induced group decreased significantly by 43%, indicating that the intestinal mucosa was damaged. The DSF treatment could restore the reduced number of goblet cells to 72% when compared with the number in the control group (Figure 2A,B). To further investigate the LPS-induced intestinal injury caused by the DSF treatment, H&E staining was used to evaluate the histopathological changes in the zebrafish intestine. Compared with the control group, the LPS treatment caused the intestinal tissue of zebrafish embryos to loosen and the intestinal epithelial cells to fall off (Figure 2C). After the addition of the DSF, the structure of the intestinal tissue was restored to a certain extent (Figure 2C).

### 2.3. DSF Decreases the Abnormal Accumulation of Macrophages and Neutrophils in Zebrafish

**Figure 3:**
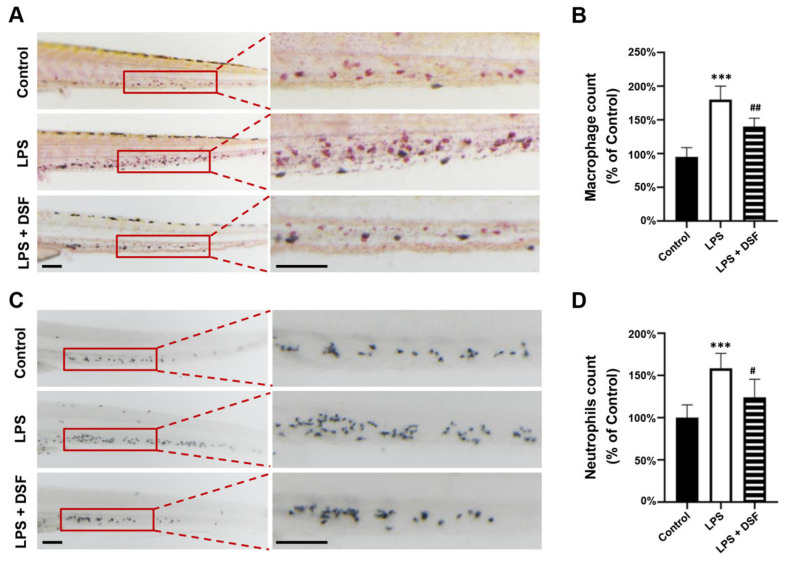
DSF prevented LPS-induced accumulation of macrophages and neutrophils in zebrafish embryos. (**A**) The macrophages were labeled with Neutral red staining. (**B**) Quantitative analysis of macrophages in the same area (red boxes) in A. (**C**) The neutrophils were labeled with Sudan Black B staining. (**D**) Quantitative analysis of neutrophils in the same area (red boxes) in C. Data are represented as mean ± S.D. *** *p* < 0.001 vs. control group; ^#^ *p* < 0.05, ^##^ *p* < 0.01 vs. LPS group. Scale bar, 100 μm.

The zebrafish primarily relies on the innate immune system in the early stage of development. Neutrophils and macrophages are the main components of the zebrafish innate immune system. Neutrophils can rapidly gather at the site of injury and respond to inflammation, while macrophages can phagocytose pathogens and tissue debris. The transparency of the zebrafish larvae allows for the real-time visualization of inflammatory cell migration in vivo [22,23]. The LPS-induced inflammatory response resulted in the abnormal aggregation of macrophages (Figure 3A) and neutrophils (Figure 3C) in the zebrafish tail region, and the number of macrophages and neutrophils also increased to 190% and 160%, respectively, compared with the numbers in the control group. The DSF treatment significantly reduced the increased number of macrophages and neutrophils to 147% and 121%, respectively (Figure 3B,D). This suggests that the addition of DSF can reduce the abnormal migration and accumulation of macrophages and neutrophils in zebrafish embryos.

### 2.4. DSF Suppresses the Production of ROS and the High Expression of Inflammatory Factors in Zebrafish

**Figure 4:**
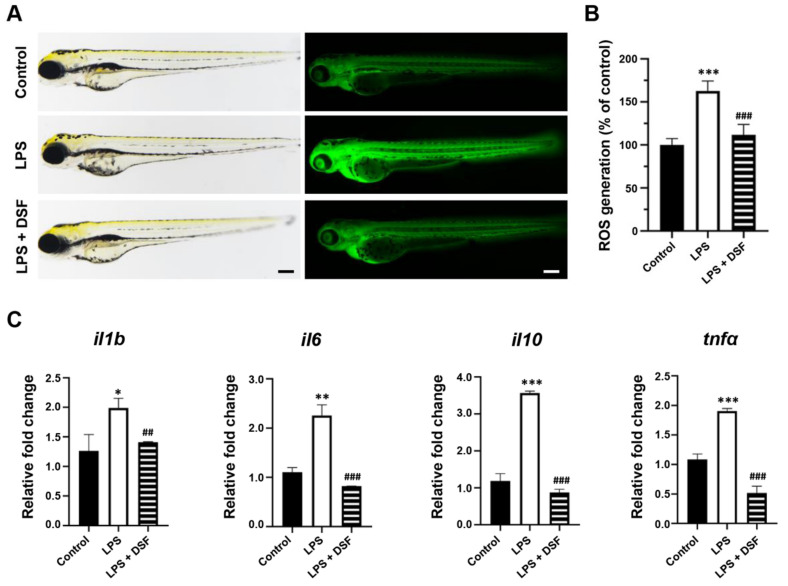
DSF inhibited LPS-induced ROS production and inflammation in zebrafish embryos. (**A**) ROS generation in zebrafish embryos was detected with fluorescent probe DCFH-DA staining. (**B**) ROS levels were measured by quantification of fluorescence intensity for individual embryos using fluorescence microscopy and ImageJ analysis. (**C**) The relative mRNA expression levels of inflammatory markers (*il1b*, *il6*, *il10*, and *tnfα*) were measured with qRT-PCR. Data are represented as mean ± S.D. * *p* < 0.05, ** *p* < 0.01, *** *p* < 0.001 vs. control group; ^##^ *p* < 0.01, and ^###^ *p* < 0.001 vs. LPS group. Scale bar, 200 μm.

ROS are very important in the regulation of the immune system and are one of the key inflammatory mediators. Excessive ROS can lead to various physiological and biochemical damages and trigger inflammation [31]. The level of ROS in zebrafish can be detected with dichloro-dihydro-fluorescein diacetate (DCFH-DA) staining. Compared with the control group, the fluorescence intensity of the LPS-induced group was enhanced (Figure 4A), and the relative fluorescence intensity analysis showed that the value was 163% (Figure 4B), indicating that the production of ROS was significantly increased in the LPS inflammation group. However, the level of excess ROS production could be reduced with the DSF treatment to 112%, similar to the level of the control group (Figure 4A,B). In addition, an abnormally high expression of related inflammatory factors, such as *il1b*, *il6*, *il10*, and *tnfa*, in the LPS-induced inflammation group was restored by with the DSF treatment (Figure 4C). The result indicated that the DSF treatment could prevent the LPS-induced inflammatory response, and further highlighted the anti-inflammatory effect of the DSF on zebrafish.

### 2.5. DSF Reduces Cell Death and the Abnormal Expression of Apoptosis Markers in Zebrafish

**Figure 5:**
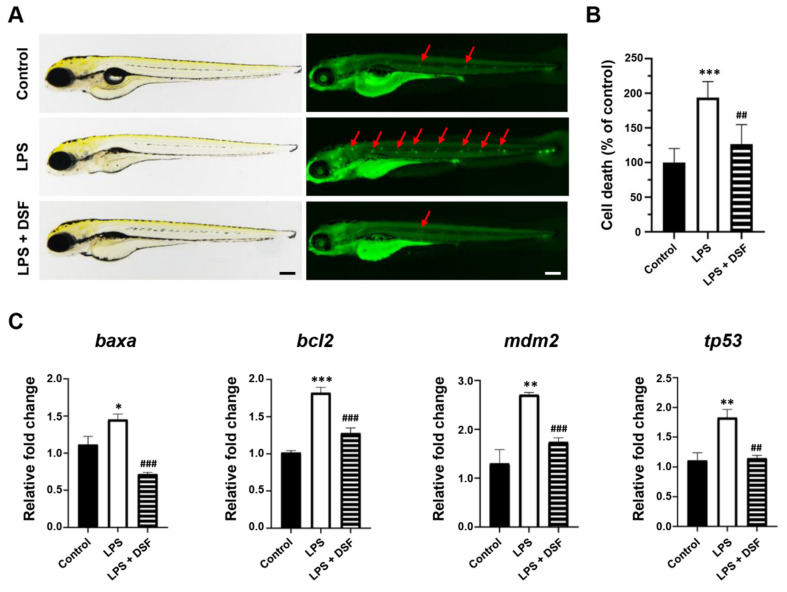
DSF reduced LPS-induced apoptosis in zebrafish embryos. (**A**) The prevalence of apoptosis in zebrafish embryos was detected with fluorescent dye AO staining; red arrows indicate the apoptotic cells. (**B**) The fluorescence intensity was quantified for individual zebrafish using ImageJ analysis. (**C**) The relative mRNA expression levels of apoptosis markers (*baxa*, *bcl2*, *mdm2*, and *tp53*) were measured with qRT-PCR. Data are represented as mean ± S.D. * *p* < 0.05, ** *p* < 0.01, *** *p* < 0.001 vs. control group; ^##^ *p* < 0.01, and ^###^ *p* < 0.001 vs. LPS group. Scale bar, 200 μm.

The accumulation of ROS can lead to progressive cell damage and, eventually, cell death. To further examine the anti-inflammatory effect of the DSF on zebrafish, an Acridine orange (AO) staining assay was carried out to evaluate the prevalence of apoptosis in zebrafish embryos treated with LPS or LPS + DSF. The AO dye could bind to the condensed chromatin in the nucleus, and apoptotic cells showed a bright green signal in LPS-induced embryos (Figure 5A, red arrow). However, the LPS-induced cell impairment was mitigated by the DSF treatment (Figure 5A). In addition, the quantitative analysis of the zebrafish embryos stained with AO clearly showed that, compared with the control group, the apoptosis rate of the LPS-induced group increased dramatically to 194%, which could be significantly reduced to 127% after the DSF treatment (Figure 5B).

Furthermore, the expression levels of apoptosis-related markers *baxa*, *bcl2*, *mdm2*, and *tp53* in zebrafish were significantly increased after the LPS treatment. When the DSF was added, the expression levels of these abnormally highly expressed genes tended to return to normal (Figure 5C).

### 2.6. Transcriptome Analysis of Zebrafish Embryos Treated with DSF

**Figure 6:**
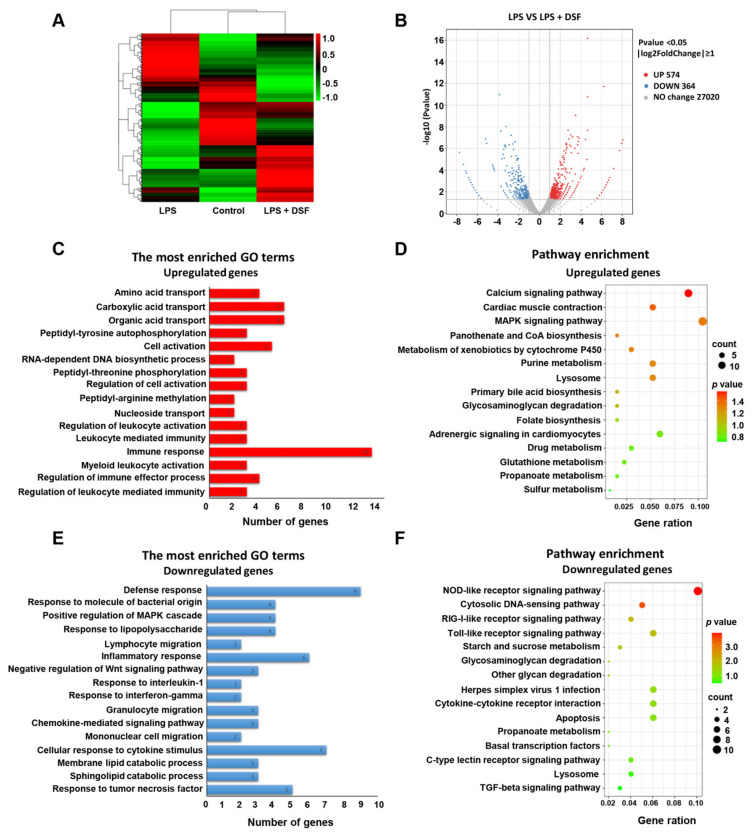
Transcriptome analysis of differentially expressed genes between LPS and LPS + DSF groups. (**A**) Heat map representation showing a total of 2029 DGEs in control, LPS- and LPS + DSF-treated zebrafish embryos. (**B**) Volcano plots show a total of 938 DGEs in LPS and LPS + DSF groups. (**C**) GO term analysis and (**D**) KEGG pathway analysis for up-regulating DEGs in LPS + DSF zebrafish embryos compared to the LPS group. (**E**) GO term analysis and (**F**) KEGG pathway analysis of down-regulated DEGs in LPS + DSF zebrafish embryos compared to the LPS group. The size of the dot indicates the number of genes enriched in an individual item. The color of the dot represents the *p* value.

To explore the underlying molecular mechanism of the anti-inflammatory effect of the DSF on zebrafish, the total RNA was extracted from three groups of zebrafish embryos at 96 hpf, including the control, LPS, and LPS + DSF, respectively, followed by an RNA-Seq analysis. After removing reads containing the adapter, base information not possible to determine, and low-quality raw data, 96.46, 96.97, and 96.52% clean reads in the three groups were mapped to the reference genome (Danio rerio: NCBI_GRCz11), correspondingly (Table S1). A total of 2029 DEGs was annotated from all the comparison groups, the FPKM value of the gene was clustered using mainstream hierarchical clustering, and the row (row) was normalized (Z-score) (Figure 6A). After the quality screening, a total of 938 DEGs was identified in the LPS and LPS + DSF groups, (|log2(FoldChange)|≥ 1, *p* < 0.05). Among these genes, 574 genes were up-regulated and 364 genes were down-regulated (Figure 6B).

To further explore whether this altered transcriptional profile was associated with any biological processes or specific pathways, GO enrichment and a KEGG pathway analysis were performed. It was found that the up-regulated genes were mainly related to the regulation of the immune response, immune effector process, and leukocyte-mediated immunity (Figure 6C, Table S2). In addition, through the KEGG analysis, it was found that the up-regulated genes were enriched in the calcium signaling pathway, lysosome, and the MAPK signaling pathway (Figure 6D, Table S2). The down-regulated genes were mainly involved in the inflammatory response, cellular response to cytokine stimulus, response to lipopolysaccharide, and response to tumor necrosis factor (Figure 6E, Table S3). In the KEGG analysis, these genes were mainly involved in the Toll-like receptor signaling pathway, cytokine–cytokine receptor interaction, lysosome, and apoptosis (Figure 6F, Table S3). These findings suggest that the molecular mechanism of the anti-inflammatory effect of the DSF in LPS-induced inflammatory injury may be achieved by influencing a series of inflammatory signaling factors, including the Toll-like receptor signaling pathway.

### 2.7. DSF Inhibits LPS-Induced Inflammation through Toll-like Receptor Signaling

**Figure 7:**
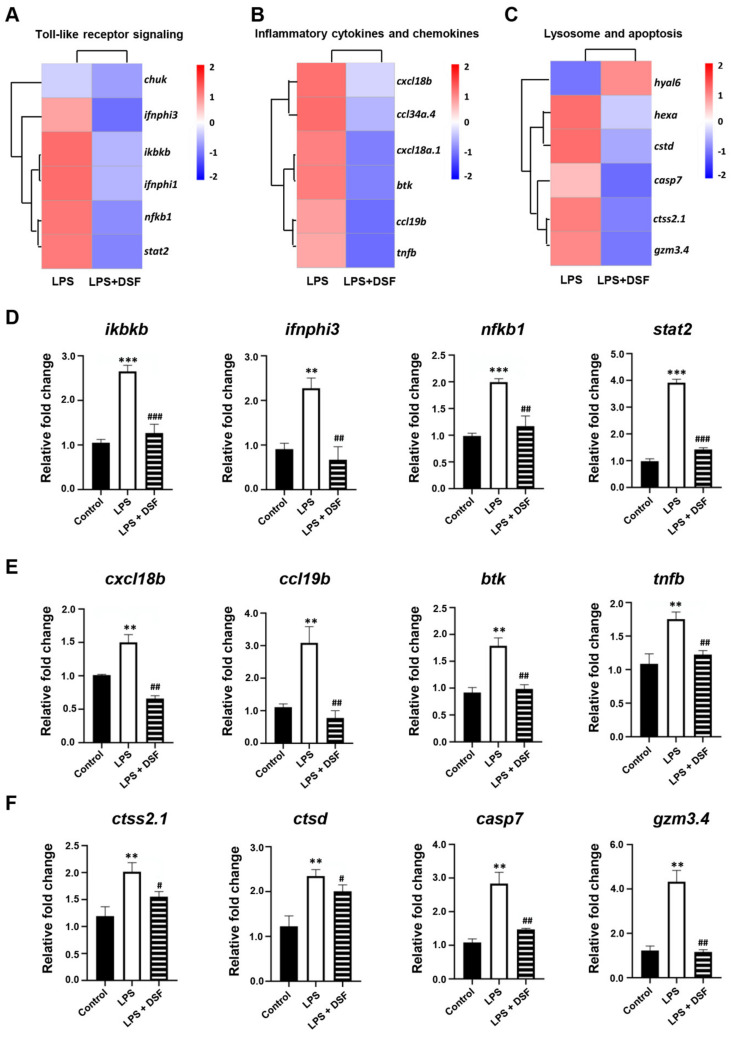
DSF targeting downstream signaling screening and verification. (**A**) Toll-like receptor signaling, (**B**) inflammation cytokines and chemokines, and (**C**) lysosome-apoptosis-related genes explored with heat map analysis and verified with qRT-PCR (**D**–**F**). Data are represented as mean ± S.D. ** *p* < 0.01, *** *p* < 0.001 vs. control group; ^#^ *p* < 0.05, ^##^ *p* < 0.01, and ^###^ *p* < 0.001 vs. LPS group.

**Figure 8:**
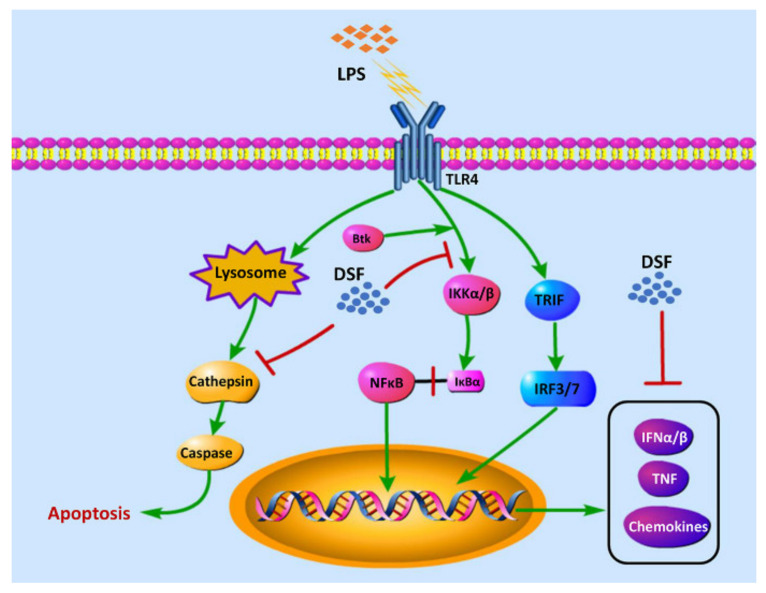
The hypothesis of the molecular mechanism of DSF in the anti-inflammatory effect.

Our previous studies showed that the treatment of the DSF could prevent the inflammatory response and reduce cell death in zebrafish in vivo (Figure 4 and Figure 5). According to the RNA-Seq data obtained here from zebrafish, it was found that the expression of genes involved in Toll-like receptor signaling, inflammation chemokines, and lysosome-apoptosis-related genes was changed in the DSF + LPS group compared with the LPS group (Figure 7A–C). The subsequent qRT-PCR validation in zebrafish whole embryos further revealed that the abnormal expression of Toll-like receptor signaling (*ikbkb*, *ifnphi3*, *nfkb1*, and *stat2*) and related inflammation cytokines and chemokines (*cxcl18b*, *ccl19b*, *btk*, and *tnfb*) in the LPS group was significantly restored to normal expression levels in varying degrees after the DSF treatment when compared with the expressions of these genes in control embryos (Figure 7D–E). Meanwhile, when detecting the expression of lysosome-apoptosis-related genes, such as the lysosomal acid hydrolases *ctss 2.1* and *ctsd*, which are related to the synthesis of cathepsin and participate in the apoptotic process [32,33], *gzm3.4*, which encodes a protein that can activate the caspase chain reaction and lead to apoptosis [34,35], and *casp7*, from a family of protease enzymes playing important roles in programmed cell death [36], the abnormal expression of these genes was restored in the DSF + LPS group compared with the LPS group (Figure 7F). These findings elucidated the potential mechanism of DSF in the treatment of LPS-induced inflammation and apoptosis by inhibiting the activation of signaling in the Toll-like receptor pathway, attenuating the expression of pro-inflammatory cytokines and chemokines, and regulating the activation of the caspase cascade by restoring the expression of lysosomal cathepsins and apoptosis signaling (Figure 8).

## 3. Discussion

The DSF is a *cis*-2-unsaturated fatty acid that functions as a quorum-sensing signal molecule in *Xcc*. The DSF has been studied to mediate interspecies and interkingdom communication between microorganisms and between parasitic bacteria and hosts. It has been reported that the DSF can trigger a range of innate immune responses, such as allergic response (HR)-like responses, programmed cell death, the accumulation of autofluorescent compounds, production of hydrogen peroxide, and expression of pathogenesis-related protein 1 (PR1) in *Arabidopsis*, *N.benthamiana*, and rice models [37]. In animal pathogens, DSF family signals can affect the expression of virulence factors. In HeLa cells and zebrafish infection models, exogenous BDSF (*cis*-2-dodecenoic acid) significantly reduced the cytotoxicity and mortality of *P. aeruginosa* in infected fish [38]. In the mouse colitis model, the DSF c2-HAD inhibited the expression of the virulence genes of intestinal pathogens and reduced the colonization of pathogenic bacteria in the intestine by interacting with transcription regulators of the AraC family [19]. In addition, the DSF can regulate the formation of a bacterial biofilm and reduce the tolerance of pathogenic bacteria to antibiotics during paired antibiotic administration [18]. However, research on the biological activity of the DSF in animals is very limited.

In this study, we investigated the anti-inflammatory activity of the DSF and its potential molecular mechanism using a zebrafish inflammation model. Through a series of in vivo analyses, we found that the DSF treatment could alleviate intestinal injury, reduce the abnormal migration and aggregation of inflammatory cells (neutrophils and macrophages), suppress the production of excessive ROS, and eliminate apoptosis.

Furthermore, through an RNA-Seq analysis, 938 DEGs were screened with 574 up-regulated genes and 364 down-regulated genes between LPS and LPS + DSF treatment zebrafish embryos. Through GO and KEGG analyses, multiple affected inflammatory pathways were found in zebrafish treated with LPS and LPS + DSF, among which Toll-like receptor signaling was identified as one of the important signaling pathways for the DSF to prevent the abnormal activation of inflammatory factors and alleviate inflammation.

TLRs are a family of membrane-bound receptors that serve as representatives of pattern recognition receptors [39]. TLRs are involved in the recognition of pathogen-associated molecular patterns (PAMPs) by the innate immune system in microorganisms [40]. TLR4 is the main LPS receptor, which activates and triggers MyD88 and TRIF-dependent signaling pathways under LPS stimulation, and promotes the expression of downstream inflammatory factors and type 1 interferon [41]. IKK (IκB kinase) is located downstream of the MyD88-dependent pathway and leads to the phosphorylation of the IκB (NF-κB inhibitor) protein, which is responsible for hiding the nuclear localization domain (NLD) of NFκB [42]. After the ubiquitination and degradation of IκB, transcription factor NF-κB is able to transfer from the cytoplasm into the nucleus, thus, participating in the inflammatory response process [40]. Our studies found that the DSF was able to inhibit the activation of NFκB signaling (*nfkb*) by reducing the expression of IKK-related genes (*ikbkb*), which eventually led to a decrease in the expression of pro-inflammatory cytokines (*tnfa*, *tnfb*, *il1b*, *il6*, and *il10*). In the TRIF-dependent signaling pathway, the DSF decreased the expression of IFNα (*ifnphi3*) and affected the activation of STAT signaling (*stat2*). Type I interferon (IFN-α/β), an important part of the innate immune response, can enhance the immune response to suppress viral as well as pathogenic agents [43]. STAT2 plays a key role in the expression of ISGs (interferon stimulated genes) in response to IFN stimulation [44].

In addition, the expression of a set of cytokines and chemokines downstream of Toll-like signaling was also restored with the DSF treatment. When inflammation occurs, inflammatory cells overexpress pro-inflammatory cytokines, chemokines, and adhesion molecules [45]. Among them is Bruton’s tyrosine kinase (btk), which responds to LPS stimulation by regulating the polarization of macrophages, the activation of NF-κB and IRF3, and the production of IFN through the TLR signaling [46]. Chemokines can act as chemotactic cytokines to activate and regulate the migration and location of immune cells in infected or damaged organs or tissues [47]. The function of cxcl18b is similar to that of Cxcl8a/Interleukin-8 in humans and has chemotaxis to neutrophils [48]. The CC chemokine ligand 19b (ccl19b) also has the function to coordinate the migration of macrophages [49]. Our data in zebrafish suggested that the DSF inhibited the recruitment of neutrophils and macrophages to sites of inflammation by suppressing the expression of chemokines.

With the RNA-Seq screening in the LPS and LPS + DSF zebrafish embryos, genes related lysosomes were revealed to be enriched with the KEGG analysis. Lysosomes are membrane-bound organelles present in animal cells that participate in both classical and non-classical autophagy processes, and affect inflammatory responses [50]. As the center of cell recycling, lysosome contains a large number of acidic hydrolases, which can degrade most cell macromolecules [51]. Lysosomal membrane permeability and the subsequent leakage of lysosomal contents into cytoplasmic lysis led to the so-called lysosomal cell death. This form of cell death is mainly carried out by lysosomal cathepsins [52]. Cathepsin S (CTSS) belongs to the L-like cathepsin subfamily of cysteine proteases. In the process of the inflammatory response, CTSS is highly expressed in many immune cells and is considered to play a key role in the immune response after bacterial infection [53]. Under LPS stimulation, the expression of *ctss* increased. Lysosome rupture accelerates the diffusion of CTSS to the cytoplasm and increases cell death [32]. Cathepsin D (CTSD) is an aspartic protease, which is involved in lysosomal digestion, protein synthesis, and activation [33]. It is also related to the immune response to infection. Mature cathepsin D regulates intrinsic apoptotic pathways by stimulating the release of cytochrome c (CytC) from mitochondria [54]. Studies have shown that CTSS and CTSD can induce apoptosis by promoting the cleavage and activation of the caspase family after entering the cytoplasm [55]. Our analysis displayed that the DSF could significantly inhibit the abnormal up-regulation of *ctss 2.1* and *ctsd* expression in a zebrafish embryo inflammation model. It suggested that the inhibitory effect of the DSF on apoptosis might be due to the reduction in cathepsin leakage due to an impaired lysosomal membrane permeability. As a quorum-sensing signaling molecule, it has been pointed out that type I autoinducers represented by N-acyl homoserine lactone (AHL) can interact with inflammatory pathways and affect the innate immune system [56]. However, the DSF and its effects on immune cells, the regulation of inflammatory pathways, lysosomal enzymes transport, and potential receptors still need to be further explored.

Bacteria and their eukaryotic hosts have co-evolved for millions of years, and the former learned how to intercept eukaryotic signaling systems for the successful colonization of the host [57]. The findings in the present study raised the question of whether the DSF-mediated interkingdom communication between bacteria and zebrafish naturally occurs in the growth and development of zebrafish. The DSF-producing genus *Burkholderia* was found in the gut of wild and laboratory mice using metagenomic analyses [58]. *Stenotrophomonas maltophilia* was found to be a constituent of the crypt-specific core microbiota of the murine colon [59]. Diverse bacterial species have been identified in the zebrafish gut, and they are crucial for the zebrafish metabolism, intestinal development, and the general evolution of intestinal ecosystems [60]. Thus, future research is required to identify a DSF-producing commensal or pathogenic pathogen in zebrafish and its possible interaction with zebrafish.

## 4. Materials and Methods

### 4.1. Ethics Statement

The zebrafish experiments were conducted according to the ethical guidelines of Northwest University. All experimental protocols were approved by the Experimental Animal Management and Ethics Committee of Northwest University, and the ethical code was NWU-AWC-20211101Z.

### 4.2. Preparation of DSF

The preparation of DSF was previously described by He et al. Briefly, the *Xcc* strain was cultured in nutrient agar (NA) medium (5 g L^−1^, 3 g L^−1^ beef extract, 10 g L^−1^ sucrose, and 1 g L^−1^ yeast extract, pH 7.0) for 48 h (hours). The supernatant of bacteria was collected using centrifugation at 3800 rpm for 30 min at 4 °C (J6-HC Centrifuge, BECKMAN COULTER™, Brea, CA, USA), and the pH of the supernatant was adjusted to 4.0. Extraction was carried out twice by adding an equal volume of ethyl acetate. Then, the ethyl acetate fraction was collected and the solvent was removed through rotary evaporation at 40 °C until it was dried. The residue was dissolved in methanol. The crude extract was subjected to flash column chromatography using a silica gel column (12 × 150 mm, Biotage Flash 12 M cartridge, Biotage, Uppsala, Sweden), and eluted with ethyl acetate-hexane (25:75, *v*/*v*, 0.05% acetic acid). The crude DSF was then collected, evaporated, and dissolved in methanol for high-performance liquid chromatography (HPLC) (Agilent, Palo Alto, CA, USA) analysis with a C18 reverse-phase column (Zorbax XDB; 5 μm, 4.6 × 150 mm). The purified DSF was collected, condensed, and quantitatively analyzed using the method of He et al. [61].

### 4.3. Zebrafish Maintenance and Embryo Collection

Adult AB wild-type zebrafish were cultured in a circulating water system with a 14 h light/10 h dark cycle and were fed three times per day. Female and male zebrafish were placed in the breeding tank at a ratio of 1:1 and separated by a divider. Embryos were collected the next morning after the divider was removed [62]. The healthy fertilized eggs were cultured in an embryo culture medium at 28.5 °C, and staged according to Kimmel et al. [63].

### 4.4. Measurement of the Toxicity of DSF on Zebrafish Embryos

To evaluate the toxicity of DSF, a dose–response analysis was carried out to determine the median lethal dose (LC_50_). The zebrafish embryos (30 for each concertation) were treated for five days from 24 hpf (hours post fertilization) to 120 hpf, with seven different concentrations (10, 20, 30, 40, 50, 75, and 100 μM) of DSF. Embryos treated with embryo culture medium were used as controls. The solution was changed every 24 h. The survival rate was determined every day by counting the hatched embryos that survived. Malformation was measured from 72 hpf to 120 hpf. Embryos at 72 hpf were photographed under an SMZ25 stereomicroscope with a DS-Ri2 digital camera (Nikon, Tokyo, Japan). The body lengths of 72 hpf embryos were analyzed and recorded using ImageJ software. Each experiment was performed three times with three replicates for each concentration. In subsequent experiments, a concentration (20 μM) with no apparent embryo toxicity was chosen.

### 4.5. Construction of LPS-Induced Inflammation Zebrafish Model

Normally developed embryos at 8 hpf were transferred into 24-well plates with 15 embryos per well and three replicates were set for each group. Three experimental groups were set up: control group, LPS group, and LPS + DSF group. The LPS group was treated with LPS at a concentration of 25 μg/mL [64], in order to generate a zebrafish inflammation model. For the LPS + DSF group, the embryos were treated with 20 μM DSF for a 1 h pre-protect period, and then stimulated with 25 μg/mL LPS to induce inflammation. All embryos were cultured in a constant temperature incubator at 28.5 °C until 72 h, 96 h, or 120 h, and the medium was replaced every 24 h.

### 4.6. Intestinal Histopathology

To assess the histopathological changes of the zebrafish intestine, embryos at 120 hpf were fixed in 4% paraformaldehyde (PFA) overnight at 4 °C. After fixation, the embryos were embedded in paraffin after sanguinarine treatment and cut into 5 μm sections. The paraffin sections were stained with hematoxylin–eosin (H&E) for histopathological assessment under an upright microscope with a digital camera (Nikon, Tokyo, Japan).

### 4.7. Alcian Blue Staining

To visualize the acidic mucin produced by goblet cells in the zebrafish intestine, larvae at 120 hpf were fixed in 4% PFA overnight at 4 °C. Larvae were rinsed in acidic ethanol (70% ethanol with 1% concentrated hydrochloric acid), and incubated in Alcian blue staining solution (0.1% Alcian blue in acidic ethanol; Sigma, St. Louis, MO, USA) for 3 h at room temperature or overnight at 4 °C. Embryos were rinsed repeatedly with acidic ethanol and stored in glycerol for photography.

### 4.8. Macrophage Neutral Red (NR) Staining and Neutrophil Sudan Black B (SB) Staining

Neutral red (NR) is a dye that can be used to stain living cells or tissues. Generally, NR can be phagocytized by lysosomes and enriched in phagocytes. Due to the strong phagocytic activity of macrophages, NR dye can be used in vivo to label macrophages in zebrafish. The method was as follows: embryos at 72 hpf were stained with NR staining solution (Solarbio, Beijing, China) at room temperature in the dark for 5 h. Stained embryos from the NR staining were cleared in PBS and photographed under a Nikon SMZ25 microscope system.

Sudan Black B (SB) is a lipid-soluble dye that stains lipids in the cytoplasm of neutrophils in vivo. For SB staining, zebrafish embryos were fixed in 4% PFA at 4 °C overnight. After rinsing with PBST (0.1% Tween-20 in PBS) three times, embryos were stained with SB staining solution for 30 min in the dark at room temperature. Stained embryos were cleared with 70% ethanol and washed with PBST. Photography was carried out under Nikon SMZ25 microscope system. The SB staining solution was prepared according to the method previously described by Zhang et al. [64].

### 4.9. Measurement of ROS Production

The production of intracellular ROS in zebrafish embryos was detected using an oxidation-sensitive fluorescent probe dye, dichloro-dihydro-fluorescein diacetate (DCFH-DA), according to the reported method [65]. Briefly, embryos at 72 hpf were placed into 6-well plates and the DCFH-DA probe (Beyotime, Shanghai, China) was added with a final concentration of 20 μg/mL. The embryos were incubated in the dark for 1 h at 28.5 °C for ROS detection and then washed with a clean embryo culture medium. The stained embryos were imaged under a fluorescence microscope with a DS-Ri2 digital camera (Nikon, Tokyo, Japan). The fluorescence intensity of the embryo was quantified with ImageJ software (NIH, Washington, DC, USA).

### 4.10. Acridine Orange (AO) Staining

Acridine orange (AO) is a fluorescent dye used to detect apoptotic cells [66]. Zebrafish larvae at 96 hpf were placed into 6-well plates, and then incubated with 10 μg/mL AO staining solution (Beyotime, Shanghai, China) for 30 min in the dark at 28.5 °C. Following incubation, fish were rinsed using a clean embryo culture medium. The apoptotic cells were observed and recorded under a fluorescence microscope. The intensity of each green fluorescence signal was measured and analyzed using Image J software.

### 4.11. RNA-Seq and Bioinformatic Analysis

Total RNA was extracted from three groups of zebrafish embryos at 96 hpf, including control, LPS, and LPS + DSF, using the Trizol reagent (Ambion, Austin, TX, USA) following the manufacturer’s protocol. The RNA was quantified using a NanoDrop 2000 instrument. Libraries were constructed using NEBNext Ultra™ RNA Library Prep Kit for Illumina (NEB, Los Angeles, CA, USA) and subjected to high-throughput sequencing through the Illumina novaseq 6000 platforms (NovoTech, Beijing, China). The clean reads were mapped to the reference genome (Danio rerio: NCBI_GRCz11). Differential expressed genes (DEGs) of the two groups were analyzed using the DESeq2 R package (1.10.1). The false discovery rate (FDR) was used to correct the *p* value to determine the true DEGs. For analyzing the gene expression difference between two samples, *p* value < 0.05 and|log2(Fold change)| ≥ 1 were considered significant DEGs and used for further analysis [67].

Gene ontology (GO) and KEGG pathway examinations were performed on DEGs for enrichment analysis. GO terms and KEGG analysis with corrected *p* value < 0.05 were considered to be significantly enriched [68].

### 4.12. Real-Time Quantitative PCR (qRT-PCR)

Total RNA was extracted from zebrafish embryos and mice intestinal tissues using TRIzol reagent (Ambion, Austin, TX, USA) and subjected to reverse transcription using the SuperScriptIII (Invitrogen, Carlsbad, CA, USA). qRT-PCR was carried out as described previously using SYBR FAST Universal qPCR kit (Kapa Biosystems, Boston, MA, USA), and ViiA 7 Real-Time PCR System (ABI, Foster City, CA, USA) [69]. The PCR conditions consisted of a denaturation step for 3 min at 95 °C followed by 40 cycles of 95 °C for 3 s and 60 °C for 20 s. Primer sequences are listed in Table S4.

### 4.13. Statistical Analysis

Each experiment was repeated at least three times. Group data were analyzed with one-way analysis of variance (ANOVA), followed by Student’s *t*-test, using the GraphPad Prism 5.0 software (GraphPad Software, San Diego, CA, USA). Results were expressed as means ± SD. Values of *p* < 0.05 were considered statistically significant.

## 5. Conclusions

In conclusion, using an LPS-induced zebrafish inflammation model, we demonstrated that the DSF could alleviate intestinal injury, eliminate apoptosis, and reduce inflammation in a vertebrate model. Transcriptome and bioinformatics analyses further revealed that the possible mechanism of the anti-inflammatory effect of the DSF might be through Toll-like receptor signaling to attenuate the expression of inflammatory factors and lysosome-mediated apoptosis (Figure 8). Our results, for the first time, confirmed the anti-inflammatory activity of the DSF in a zebrafish model, and suggested a potential application of the DSF in the pharmaceutical industry.

## Data Availability

Not applicable.

## Supplementary Materials

The following supporting information can be downloaded at: https://www.mdpi.com/article/10.3390/ijms23137110/s1.
