# Supplementary material for: Bacterial Quorum-Sensing Signal DSF Inhibits LPS-Induced Inflammations by Suppressing Toll-like Receptor Signaling and Preventing Lysosome-Mediated Apoptosis in Zebrafish
Source: Int J Mol Sci. 2022 Jun 26;23(13):7110. doi: 10.3390/ijms23137110 (PMC9266882; doi:10.3390/ijms23137110)
Supplement: Supplementary file 1 [file ijms-23-07110-s001.zip › Suppl. Table S4.pdf]

Supplementary Table S4: List of all primer sequences

Zebrafish cDNA primer sequence used for Q-PCR

Gene	Primer	Sequence
tnfa	forward	AAGGAGAGTTGCCTTTACCG
	reverse	GCCTTGTGAAATGCGATCTCT
il10	forward	GCTCTGCTCACGCTTCTTC
	reverse	CCAAGTCATCGTTGGACTCATA
il6	forward	TGCTACACTGGCTACACTCTT
	reverse	CACATCCTGAACTTCGTCTCC
il1b	forward	ATGGCGAACGTCATCCAAGAG
	reverse	TTCAAGTCGCTGCTTCCGGCT
bcl2	forward	TCAATAAAGCAGTGGAGGAATC
	reverse	TCAAATGAGGGTCTGAACGAG
tp53	forward	CCAGCAGCGATGAGGAGAT
	reverse	GCAGGCACCACATCACTTAA
baxa	forward	TTGTGTATGAGCGTGTTCGT
	reverse	AGTCGGCTGAAGATTAGAGTTG
mdm2	forward	CTCTCGCTCATCTACCTCAC
	reverse	CCAATCACGCACCAAGACAG
ccl19b	forward	TGTGGAGGACAGGAGAAGAGAC
	reverse	CGGTCGCTGGTGGTTAAACA
cxcl18b	forward	CTGCTGCTCGCGGTAGTTTA
	reverse	TCAACTTTGTCGCAGTTTGG
ifnphi3	forward	ACTCCAGAACATTGACGCATTG
	reverse	AACCTTGAGCGAAGCCTCTC
stat2	forward	GGAGGAGCAGCAGGATGAGT
	reverse	CGTCCAGCATAGCCGAGATT
ctss2.1	forward	GGTGCTTGTGGTTCTTGTTGG
	reverse	CATACTGGAAGGCGTCACTCAT

ctsd	forward	CCTATCCTCGCATCGCTGTC
	reverse	GCCTGTCTGCTGATGTCCAC
caspase7	forward	ACGGTGAAGAAGGTGTTGTGTT
	reverse	GCAAGCCTGGATGAGGAAGAG
nfkb1	forward	CCAGCCATCCACAACAATAACA
	reverse	GGTAGGACTTGCGGTTCTTCT
ikbkb	forward	ACCAGCATTCAGATCGCCATC
	reverse	TCTCGCCGCAACCACATTCAT
btk	forward	GCACCAATGACTACCAATGACT
	reverse	TGACTGCGGTTGATGTTCTTG
tnfb	forward	TGTGCGTTGAAGATGTTGAAGG
	reverse	TAGAGACTGGCAGACGGAATTG
gzm3.4	forward	CAGGAGGGATGGAGAGTGGTAT
	reverse	CAACAATGAGCCGAAGTCAACA
actin	forward	GGCTACAGCTTCACCACCA
	reverse	TGCTGATCCACATCTGCTG
